# Electronic Health Record Portal Adoption: a cross country analysis

**DOI:** 10.1186/s12911-017-0482-9

**Published:** 2017-07-05

**Authors:** Jorge Tavares, Tiago Oliveira

**Affiliations:** NOVA IMS, Universidade Nova de Lisboa, Campus de Campolide, 1070-312 Lisbon, Portugal

**Keywords:** UTAUT2, Technology adoption, eHealth, Healthcare consumers, Electronic Health Records

## Abstract

**Background:**

This study’s goal is to understand the factors that drive individuals to adopt Electronic Health Record (EHR) portals and to estimate if there are differences between countries with different healthcare models.

**Methods:**

We applied a new adoption model using as a starting point the extended Unified Theory of Acceptance and Use of Technology (UTAUT2) by incorporating the Concern for Information Privacy (CFIP) framework. To evaluate the research model we used the partial least squares (PLS) – structural equation modelling (SEM) approach. An online questionnaire was administrated in the United States (US) and Europe (Portugal). We collected 597 valid responses.

**Results:**

The statistically significant factors of behavioural intention are performance expectancy ($\widehat{\beta}$_total_ = 0.285; *P* < 0.01), effort expectancy ($\widehat{\beta}$_total_ = 0.160; *P* < 0.01), social influence ($\widehat{\beta}$_total_ = 0.198; *P* < 0.01), hedonic motivation ($\widehat{\beta}$_total_ = −0.141; *P* < 0.01), price value ($\widehat{\beta}$_total_ = 0.152; *P* < 0.01), and habit ($\widehat{\beta}$_total_ = 0.255; *P* < 0.01). The predictors of use behaviour are habit ($\widehat{\beta}$_total_ = 0.145; *P* < 0.01), and behavioural intention ($\widehat{\beta}$_total_ = 0.480; *P* < 0.01). Social influence, hedonic motivation, and price value are only predictors in the US group. The model explained 53% of the variance in behavioural intention and 36% of the variance in use behaviour.

**Conclusions:**

Our study identified critical factors for the adoption of EHR portals and significant differences between the countries. Confidentiality issues do not seem to influence acceptance. The EHR portals usage patterns are significantly higher in US compared to Portugal.

**Electronic supplementary material:**

The online version of this article (doi:10.1186/s12911-017-0482-9) contains supplementary material, which is available to authorized users.

## Background

Our study centres on a particular type of eHealth technology, the electronic health record (EHR) portals, also called EHR patient portals [1–4]. We can define an EHR portal as a web based application that combines an EHR system and a patient portal [1, 2, 5]. EHR portals support patients in managing their own activities, thus making the use of the healthcare system more effective, not only from the patient care perspective, but also from the financial standpoint, due to increasing healthcare costs in several countries [6–9]. Several authors have studied the impact of cultural influences in the adoption of eHealth patient- focused technologies as well the effect of specific moderators [10–12]. Our study analyses the impact on EHR portals adoption of different healthcare systems, by using two countries that use completely different approaches [13]. The first is the national health system (NHS) model that features universal coverage, with funding from general tax revenues and public ownership of the health infrastructure, and in our study is represented by Portugal [13]. The other is the private health insurance (PHI) model coverage that is based on private insurance only, which is also the major funding source. Delivery is characterized by private ownership and in our study is represented by the United States (US) [13].

Concerns over the confidentiality of EHR have been reported in the US, where the data in an EHR regarding a patient is currently owned by the practitioner gathering the information and/or the insurance payer covering the patient [5]. Not only may the concerns about the information inside EHR be used to increase the cost of a patient health insurance in a PHI model [1, 5, 13, 14], but also the patient’s perception of the price and cost of the health services is different in an NHS versus a PHI model [1, 5, 13, 14], and deserves to be evaluated if it also affects the adoption of EHR portals differently [1]. In both the US and Europe governments seek to promote the spread and use of EHR portals [1].

A new guidance called “Stage 2 meaningful” use was issued by the Center for Medicare & Medicaid Services (CMS) in the US [1, 3]. It requires that the eligible professionals and healthcare facilities that take part in Medicare and Medicaid EHR incentive program must provide their patients secure online admission to their health information, including EHRs, and prove to the government that the patients are using them effectively [1, 3]. In Europe, in addition to the usual healthcare providers (such as clinics and hospitals) that provide EHR portals, governmental institutions also make these platforms available to patients [1, 6, 15]. Specifically in Portugal, the use of EHRs portals is an initiative promoted by the Portuguese government that is part of a broader e-government strategy that aims to facilitate services and communications between public services and the citizens [1]. The most important initiative is the “SNS Portal” (NHS Portal), a national EHR portal created by the Ministry of Health that allows all Portuguese citizens to schedule appointments with their general practitioner, obtain electronic medical prescriptions, access their medical records and exams results, and share information with healthcare providers [1]. Recent reports point out that stage 2 meaningful use has stimulated adoption of EHRs in the US [16], but the same findings have not been confirmed in Portugal [1]. According to the literature, adoption and continued use of a new Information Technology (IT) in general, but also in healthcare, represent different behavioural intentions [5, 17, 18]. IT adoption is the initial use of a new IT, whereas IT usage is the subsequent continued use of a new or innovative IT [5, 17, 18]. It would be interesting to verify if there are differences in the frequency of usage patterns between the two countries.

The aim of this study is to unveil a set of determinants in the adoption of EHR portals by healthcare consumers to determine if there are differences between the two countries (Portugal and the US), which we are using to represent different healthcare systems. With this purpose we suggest a new research model based on the extended Unified Theory of Acceptance and Use of Technology (UTAUT2) in a consumer context, by integrating it with the Concern for Information Privacy (CFIP) framework.

### Literature review

Several models developed from theories in sociology, psychology, and consumer behaviour have been used to describe technology adoption and usage [19]. The aim of the current study is to focus on the EHR Portals adoption from the viewpoint of the healthcare consumer. It is of the greatest importance to review the literature on this specific topic. The evaluation of the adoption of eHealth technologies by healthcare consumers still requires more attention and research due to the restricted number of studies published to date [1, 3, 5, 20, 21].

The most common adoption models used when studying eHealth and healthcare adoption by healthcare professionals are the Unified Theory of Acceptance and Use of Technology (UTAUT) [3, 22–24] and the Technology Acceptance Model (TAM) [3, 25, 26]. According to the literature, EHR form the core of many eHealth applications and thus the success of these dependents greatly on the EHR adoption by the healthcare professionals [27]. The importance of the UTAUT model in evaluating the adoption of EHR, has been recognized in the literature by the several studies published on this specific matter [28–32]. Venkatesh et al. [32] proposed a revised UTAUT for EHR system adoption and use by healthcare professionals. The revised model increased the explained variance of behaviour intention from 20% in the original model to 44% in the revised model, and is a positive indicator for the use of similar approaches with UTAUT2, with a focus on healthcare consumers [3, 19, 33]. In general all four core constructs have been showed to play a role in the adoption of EHR by healthcare professionals [28–32], but in the latest studies, performance expectancy is demonstrating an even greater role, showing that health care professionals are now expecting that EHR systems can increase their work efficiency [27, 29].

When assessing the studies published in the field of consumer health information technology adoption, most studies use TAM or extensions of TAM [20, 34–36]. Neither UTAUT nor TAM were designed with the consumer in mind. Preferably, we require a model developed for the consumer use context, and UTAUT2 was developed exactly with this aim, attaining very good results [19]. A recent study using a UTAUT2 extension showed its usefulness in evaluating the critical determinants for the adoption of EHR portals but did not account for the confidentiality issues, nor did it compare two different countries [3].

Table 1, Sums up some of the studies done in the field of eHealth, the theory or theories supporting the studies, the dependent variable that is being explained in the study, and the most important findings. The target population in the studies was patients [3, 5, 12, 34, 35, 37–39].

**Table 1:** eHealth adoption models

Theory	Dependent variable	Findings	Reference
TAM, integrated model (IM), motivational model (MM),	eHealth behavioural intention	▪ Users’ perceived technology usefulness (PU), users’ perceived ease of use (PEOU), intrinsic motivation (IM), and extrinsic motivation (MM) have significant positive influence on behavioural intention.	[34]
		▪ IM does not have a better performance than TAM or than MM when predicting behavioural intention.	
Elaboration likelihood model (ELM), concern for information privacy (CFIP)	EHR behavioural intention	▪ Privacy concern (CFIP) is negatively associated with likelihood of adoption.	[5]
		▪ Positively framed arguments and issue involvement create more favourable attitudes toward EHR behavioural intention.	
TAM (qualitative research)	eHealth services behavioural intention	▪ PU seemed to be relevant.	[37]
		▪ PEOU did not seem to be an issue.	
		▪ Although experience is not a TAM construct, it seemed to have influenced behavioural intention.	
TAM, plus several other constructs	Internet use behaviour as a source of information	▪ PU, concern for personal health, importance given to written media in searches for health information, importance given to the opinions of physicians and other health professionals, and the trust placed in the information available are the major predictors of use behaviour.	[38]
Personal empowerment	Internet use behaviour as a source of information	▪ There are three types of attitudes encouraging internet use to seek health information: consumer, professional, and community logic.	[39]
Extended TAM in health information technology (HIT)	HIT behavioural intention	▪ PEOU, PU, and perceived threat significantly influenced health consumer’s behavioural intention.	[35]
UTAUT2 extended model	Behavioural intention and use behaviour in EHR portals	▪ Effort expectancy, performance expectancy, habit, and self-perception are predictors of behavioural intention.	[3]
		▪ Habit and behavioural intention are predictors of use behaviour.	
TAM, Trust and Privacy	Intention to adopt e-Health	▪ PEOU, PU and trust are significant predictors.	[12]

Published studies point out that awareness of the lack of confidentiality and privacy concerns may reduce the adoption of eHealth tools by the patients and healthcare consumers [5, 40–42]. Studies focusing specifically on EHR show that patients are concerned about the privacy of their EHR [5]. In light of these findings we decided to evaluate confidentiality in the adoption of EHR Portal via the CFIP framework [43].

### Research model

We can define an EHR portal as a web based application that combines an EHR system and a patient portal [1, 44]. According to the literature most of the studies that have evaluated the adoption of patient portals, have used IT adoption models, like TAM or extended TAM; and more recently the use of UTAUT2 has also started to be implemented in patient centred e-health tools [1, 3, 12, 35, 36]. Because this model includes consumer specific constructs and EHR Portals can be regarded as a healthcare consumer specific tool, the literature review suggests their use with UTAUT2 [3, 14, 19, 33]. In the case of UTAUT, which was originally developed to explain employee technology acceptance and use, the model itself was not developed with IT consumer adoption in mind [19]. UTAUT2 includes the same four core UTAUT constructs, performance expectancy, effort expectancy, social influence, and facilitating conditions plus three new constructs that are consumer specific: hedonic motivation, price value, and habit [40].

**Fig. 1:**
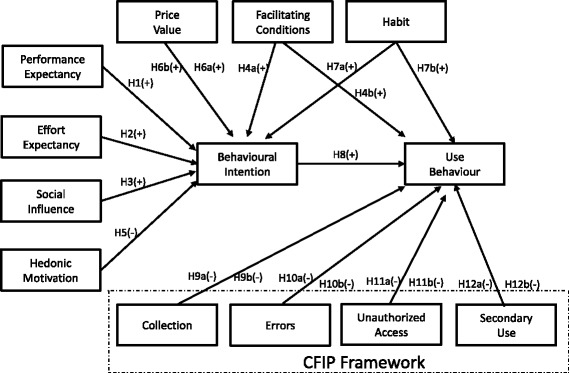
The research model

In both the US and Europe governmental initiatives are underway to incorporate patient access to their EHR via EHR Portals [1, 44, 45], and one of the most studied topics about EHR and their acceptance by the patients is the potential confidentiality concerns, which has been addressed in the literature by using the CFIP framework [5, 46]. Since an EHR Portal incorporates all the features of a Patient Portal plus the access by the patient to EHRs [1, 44], it makes sense to combine both UTAUT2 and CFIP. In the US the burden of healthcare cost is much higher to the patient due to the PHI model compared to Europe, particularly to Portugal with NHS coverage [13]. The literature review also points out that the confidentiality concerns are greater in US than in Europe, including the EHR [5, 47]. Therefore we focused our multi-group analysis approach to evaluate potential adoption differences between the two countries, by using the UTAUT2, Price Value Construct, and the CFIP framework. Fig. 1 illustrates the new research model.

Our Hypotheses *(H)* are defined according to literature findings that may regard them as non-specific to a particular health system, or specific to a particular group analysis (US and Portugal).

### UTAUT core constructs

Performance expectancy is conceptualized as the extent to which the use of a technology will provide benefits to consumers in performing specific tasks [48, 49]. Overall healthcare consumers adopt and use more eHealth technologies that deliver benefits in performing on-line health related tasks [6, 50, 51].

- *H1. Performance expectancy will positively influence behavioural intention to use*

Effort expectancy is the degree of ease related to consumers’ usage of a specific technology [48]. The easier it is for patients to grasp and use an eHealth technology, the higher is the likelihood that they will use it [6, 51].

- *H2. Effort expectancy will positively influence behavioural intention to use.*

Social influence is the degree to which consumers recognize that others who are relevant to them believe they should use a specific technology [19]. Social influence may play a substantial role in eHealth adoption, since people who share the same health concerns tend to be influenced by others having the same condition [40, 52].

- *H3. Social influence will positively influence behavioural intention to use*

Facilitating conditions refers to consumers’ perceptions of the resources and support available to perform a specific behaviour [48]. A potential obstacle to healthcare consumers’ use of eHealth services is the absence of resources or support services that allow them to access and properly use these types of platforms [51], suggesting that users with better conditions favour EHR portals adoption.

- *H4(a). Facilitating conditions will positively influence behavioural intention to use*
- *H4(b) Facilitating conditions will positively influence use behaviour.*

### UTAUT2 consumer specific constructs

Hedonic motivation is linked to the motivational principle that people approach pleasure and avoid pain [53, 54]. People use EHR portals very often when they are sick [1, 5] and that can be regarded by many as not being a pleasant process [55]. Extensive analysis has been performed in physiology and cognitive behaviour about hedonic motivation [19, 53]. Findings from literature point out that beyond the hedonic proprieties of a value target that should contribute to the engagement strength and pleasure, there are also other factors, different from the target’s hedonic proprieties, which influence engagement strength and thus contribute to the intensity of attraction or repulsion, in a manner that can be the opposite of what is expected [55]. Literature in healthcare care shows that people using more health services and e-health have greater concerns about their health, more serious health problems, and have higher depression rates than the population average [34, 55–58]. Depression and poor health are also linked to less enjoyment in life [59, 60]. Because most of the people that access EHR Portals do it because they have a health problem [1, 5], it would not be surprising that they do not regard the use as fun, because it is linked with a pre-existing health condition, and this is the factor different from the target’s hedonic proprieties that contributes to the intensity of repulsion and the decrease of enjoyment [53].

- *H5. Hedonic motivation will have a negative influence on behavioural intention to use*.

Price Value can be defined in its essence as cognitive trade-off between the perceived benefits of the applications and the monetary cost or value benefit for using them [19, 61]. In a consumer use setting, price value is an important factor since consumers must take the costs related with the acquisition of products and services [19]. If patients can obtain their exam results online via an EHR portal, they can save time and transportation costs by avoiding an uncessary trip to the clinic or hospital. US citizens that need to pay out-of- pocket or via health insurances tend to give more importance to price [1, 5, 13, 14].

- *H6 (a). Price value will positively influence behavioural intention to use*
- *H6 (b). Price value will positively influence behavioural intention to use in the US group and there will be a statistically significantly higher difference when compared with the Portuguese group.*

Habit can be conceptualized as the degree to which people tend to perform behaviours automatically because of learning [19]. Habit should positively influence eHealth adoption, since in recent studies on eHealth and EHR portals habit has shown to be a positive influencer of adoption [3, 33].

- *H7(a). Habit will positively influence behavioural intention to use*
- *H7(b). Habit will positively influence use behaviour.*

The role of behavioural intention as a predictor of use behaviour has been firmly established in eHealth, with the literature suggesting that the driver of using eHealth tools and EHR portals is preceded by the behaviour intention to use them [3, 19, 34, 35, 48, 62, 63].

- *H8. Behavioural intention will positively influence use behaviour.*

### CFIP framework

The CFIP framework was originally developed to measure beliefs and attitudes concerning individual information privacy related to the use of personal information in a business environment [43]. It was conceptualized as being composed of four dimensions: collection, errors, unauthorized access, and secondary use [43]. The CFIP framework has also been used in e-health and in the context of EHR [5, 46, 64]. Angst and Agarwal [5] found that CFIP is negatively related to the EHR adoption and Hwang et al. [64] confirmed the existence of substantial privacy concerns regarding secondary use and unauthorized access to EHRs. Overall the existing literature supports the elaboration of our hypothesis regarding CFIP [5, 46, 64]. Regarding the reasons to support the potential differences regarding confidentiality concerns between the two countries, previous international studies [47, 65] using the CFIP instrument found that consumers in countries with moderate regulatory models (e.g. the US and New Zealand) had greater privacy concerns than consumers in countries with high privacy laws regulation (e.g. the EU and more specifically Portugal) [1, 44, 62]. Related to healthcare and more specifically to EHR, existing literature also points out that patients, particularly in the US, seem to be more concerned about data privacy of their EHR records than their European counterparts [1, 5].

According to the literature the mismatch between intentions and actual behaviour is likely to arise during research on sensitive topics, such as matters related with medical areas, including access to EHR, being use behaviour a more reliable measure [5, 66]. Angst and Agarwal [5] developed their very relevant study before the meaningful use implementation, when the EHR use by patients was not at a stage of diffusion [5]. Due to this fact they measured the likelihood of adoption into the model as a means of estimating actual future behaviour [5]. Angst and Agarwal [5] stated in their paper that even if they were unable to collect actual use behavioural data, it should be a very important approach for future studies [5]. According to the scope and characteristics of our study topic, it should be useful to use a model in which we can measure actual behaviour regarding confidentiality concerns [3, 5] and UTAUT2 provides the possibility to have this theoretical contribution versus other models, like TAM, that focus on measuring behavioural intentions [67].

Collection is the concern that an extensive amount of personal information is being collected and stored in databases [40]. This concern is mentioned in the literature regarding e-health tools usage by the patients and more specifically in EHR adoption [5, 46].

- *H9 (a). Collection will have a negative influence on use behaviour.*
- *H9 (b). Collection will negatively influence use behaviour in the US group and there will be a statistically significantly higher difference when compared with the Portuguese group* [1, 5, 47]*.*

Errors are directly linked with the concern that protection against deliberate and accidental error in personal data is inadequate [43]. This concern is mentioned in the literature regarding e-health tools usage by the patients and more precisely in EHR adoption [5, 46].

- *H10 (a). Errors will have a negative influence on use behaviour.*
- *H10 (b). Errors will negatively influence use behaviour in the US group and there will be a statistically significantly higher difference when compared with the Portuguese group* [1, 5, 47]*.*

Unauthorized access is the concern that data about individuals are available to people not authorized to view or work with these data [40]. This concern is stated in the literature regarding e-health tools usage by the patients and more specifically in EHR adoption [5, 43, 61]

- *H11 (a). Unauthorized access will have a negative influence on use behaviour.*
- *H11 (b). Unauthorized access will negatively influence use behaviour in the US group and there will be a statistically significantly higher difference when compared with the Portuguese group* [1, 5, 47]*.*

Secondary use refers to the apprehension that information is collected from individuals for one purpose but is used for another secondary purpose without approval from the individuals [40]. This concern is stated in the literature regarding e-health tools usage by the patients and more precisely in EHR adoption [5, 46, 64]

- *H12 (a). Secondary use will have a negative influence on use behaviour.*
- *H12 (b). Secondary use will negatively influence use behaviour in the US group and there will be a statistically significantly higher difference when compared with the Portuguese group* [1, 5, 47]*.*

## Methods

### Measurement

The items were adopted from Wilson and Lankton [34], Venkatesh et al. [19], and Angst and Agarwal [5] with minor modifications to adapt them to EHR portals technology. The scales’ items were measured on a seven-point range scale, with a range from “strongly disagree” (1) to “strongly agree” (7). Use behaviour was measured on a different scale. The scale from UTAUT2 (from “never” (1) to “many times per day” (7)) was adjusted to “never” (1) to “every time I need” (7), since EHR portals use is not as frequent as a mobile internet use. Questions concerning, education, age and gender were also included. The questionnaire was administrated in English to the US sample and in Portuguese to the Portuguese sample, after being translated by a professional translator. Both were delivered via a web hosting. To guarantee that the content did not lose its original meaning, a back-translation was made from the Portuguese instrument to English, again done by a professional translator, and compared to the original [68]. The items are presented in detail in the Additional file 1.

In advance, before the respondents could see the questionnaire, an introduction was made describing the concept of EHR portals (Additional file 1). The purpose of this introduction was to guarantee that respondents were conscious of this concept, and had prior contact with and knowledge of EHR portals, because the lack of this prior knowledge and contact is an exclusion criterion.

### Data collection

A pilot survey was executed and we obtained 30 survey questions attesting that all of the items were reliable and valid. The pilot test survey data were not included in the main survey. The literature mentions that only a very small proportion of patients, fewer than 7%, use Patient Portals and EHR Portals [1–3, 69, 70]. Specific and suitable sampling strategies may be used to target these users, who could be regarded as a rare or low prevalence population [71, 72]. The literature mentions that users of these platforms have higher education than the population average [20, 70, 71] and as a consequence, we directed our sampling strategy to places where our target population is more concentrated [71, 72], and selected education and research institutions. This approach is supported by the literature as a valid sampling strategy for low prevalence populations [71, 72].

An email was sent between October of 2015 and February 2016, with the hyperlink of the survey, to a total of 2640 people at four institutions that provide education and research services, two of which were in Portugal and two in the US. The participants were informed by email about the study’s goal, anonymity of the information collected, and confidentiality protection. From these we obtained 276 responses in the US (21.9% response rate) and 337 responses in Portugal (24.4% response rate). Following the removal of the invalid responses, the final sample had 597 responses, 270 from the US and 327 from Portugal. An individual questionnaire was regarded invalid if not all questions were answered. According to our statistical model we cannot use unfinished or incomplete questionnaires [75, 76].

### Data analysis

In order to test the research model we used the partial least squares (PLS) – structural equation modelling (SEM), which is a variance-based method having the goal of maximizing the explained variance of the endogenous latent variables [77]. The main reasons to choose this method were the ability of PLS-SEM to handle complex models, a formatively measured construct is part of the structural model, and the fact that the PLS method is orientated to explain variance of the research model [75]. We used SmartPLS 2.0.M3 [78] software to estimate the PLS-SEM. Prior to testing the structural model we examined the measurement model to evaluate construct reliability, indicator reliability, convergent validity, and discriminant validity. For complementary statistical analysis we used SPSS 21 and SAS enterprise guide 1.3.

## Results

### Sample characteristics

The sample characteristics are shown in Table 2.

**Table 2:** Sample characteristics

	Average	Standard Deviation	
Age			
Total	33.34	10.97	*P <0.01* ^*a*^
US	36.42	11.17	
Portugal	30.80	10.13	
	Frequency	Percentage	
Gender			
Male Total	257	43.05%	*P = 0.587* ^*b*^
Female Total	340	56.95%	
Male US	120	44.44%	
Male Portugal	137	41.90%	
Female US	150	55.56%	
Female Portugal	190	58.10%	
	Frequency	Percentage	
Education			
Undergraduate Total	192	32.16%	*P <0.01* ^*b*^
Bachelor's Total	194	32.50%	
Higher than Bachelor's Total	211	35.34%	
Undergraduate US	92	34.07%	
Undergraduate Portugal	100	30.58%	
Bachelor's US	107	39.63%	
Bachelor's Portugal	87	26.61%	
Higher than Bachelor's US	71	26.30%	
Higher than Bachelor's Portugal	140	42.81%	

^*a*^ Mann–Whitney *U* test; ^*b*^ *χ* ^2^ test

Literature states that users of EHR portals are younger than the population average and have higher education [20, 73, 74], the results shown in Table 2 are in line with literature findings. Nevertheless the US sample has a slightly higher age that is statistically significant when compared with the Portuguese sample. Also regarding education, there are differences between the US and Portugal. In the Portuguese sample the percentage of respondents with higher than bachelor education is 42.81%, which is greater than the US sample with 26.30%. If we make the same analysis and compare Portugal and the US, regarding people with university degree (bachelor’s or more) versus undergraduate, there are no statistically significant differences between the groups (*P* = 0.411). Gender is not statistically different between the US and Portugal. We tested normality for the variable age for each group and the Kolmogorov-Smirnov test revealed non-normal distribution in both groups. We then proceeded with a non-parametric approach to compare the two groups.

### Usage results

Use behaviour was measured on a scale that ranges from “never” to “every time I need” (from 1 to 7). In Table 3 we see that the usage differences between the US and Portugal are all statistically significant on all features of EHR portal. These results show that the US health consumers in this sample are frequent users of EHR portals in opposition with the Portuguese sample, in which the fact that they had contact and used the technology did not make them frequent users of EHR portals [15, 79, 80].

**Table 3:** EHR Portals types of usage patterns

	Average	Median	
UB1			
Total	3.58	4.00	*P <0.01* ^*a*^
US	4.77	5.00	
Portugal	2.60	1.00	
UB2			
Total	3.97	4.00	*P <0.01* ^*a*^
US	4.84	5.00	
Portugal	3.25	2.00	
UB3			
Total	3.61	3.00	*P <0.01* ^*a*^
US	5.19	6.00	
Portugal	2.31	1.00	
UB4			
Total	3.72	4.00	*P <0.01* ^*a*^
US	5.31	6.00	
Portugal	2.41	1.00	
UB5			
Total	3.23	3.00	*P <0.01* ^*a*^
US	4.52	5.00	
Portugal	2.17	1.00	

UB1 = Management of personal information and communication with health providers; UB2 = medical appointments schedule; UB3 = Check their own EHR; UB4 = Check your medical exam results; UB5 = Request for medical prescription renewals; ^a^ Mann–Whitney *U* test

### Measurement model

The measurement model results are shown in Tables 4–8 and Additional file 2. The traditional criterion used to evaluate construct reliability, is Cronbach’s alpha (CA), which assumes that all the indicators are equally reliable, meaning that all of them have equal outer loadings on the construct [81]. In fact, PLS-SEM prioritizes the indicators according to their individual reliability [81]. For this reason the composite reliability coefficient (CR) is more appropriate for PLS-SEM, as it ranks indicators according to their individual reliability and also takes into account that indicators have different loadings, unlike CA [81]. Table 4 shows that all constructs in the three models have CR higher than 0.70, demonstrating evidence of internal consistency [75, 82].

**Table 4:** Cronbach’s alpha, composite reliability, and average variance extracted (AVE)

Construct	AVE			Composite Reliability			Cronbach’s Alpha		
	Total	US	Portugal	Total	US	Portugal	Total	US	Portugal
Behavioural Intention	0.85	0.89	0.83	0.95	0.96	0.94	0.91	0.94	0.90
Collection	0.74	0.85	0.87	0.92	0.96	0.96	0.95	0.94	0.95
Effort Expectancy	0.83	0.88	0.79	0.95	0.97	0.94	0.93	0.95	0.91
Errors	0.85	0.85	0.68	0.94	0.94	0.86	0.93	0.91	0.95
Facilitating Conditions	0.65	0.69	0.62	0.88	0.90	0.86	0.81	0.85	0.79
Habit	0.62	0.70	0.67	0.83	0.87	0.86	0.74	0.82	0.75
Hedonic Motivation	0.88	0.88	0.88	0.96	0.96	0.96	0.93	0.93	0.93
Performance Expectancy	0.85	0.89	0.83	0.94	0.96	0.94	0.91	0.94	0.90
Price Value	0.90	0.90	0.89	0.96	0.97	0.96	0.94	0.95	0.94
Secondary Use	0.71	0.78	0.73	0.91	0.93	0.92	0.89	0.90	0.88
Social Influence	0.95	0.93	0.96	0.98	0.98	0.98	0.97	0.96	0.98
Unauthorized access	0.83	0.82	0.89	0.94	0.93	0.96	0.92	0.89	0.94

**Table 5:** Correlations and square roots of AVEs in the total model

	BI	CL	EE	ER	FC	HT	HM	PE	PV	SU	SI	UA	UB
BI	0.92												
CL	−0.06	0.86											
EE	0.45	−0.17	0.91										
ER	0.04	0.00	0.23	0.92									
FC	0.40	−0.11	0.68	0.26	0.81								
HT	0.53	0.07	0.26	−0.10	0.23	0.79							
HM	0.27	−0.04	0.39	0.08	0.27	0.47	0.94						
PE	0.57	−0.08	0.50	0.24	0.42	0.38	0.39	0.92					
PV	0.49	−0.03	0.39	0.09	0.35	0.44	0.33	0.41	0.95				
SU	0.09	−0.03	0.28	0.51	0.37	−0.12	0.03	0.22	0.11	0.84			
SI	0.51	0.08	0.19	−0.11	0.20	0.57	0.28	0.36	0.37	−0.10	0.97		
UA	0.11	0.03	0.31	0.69	0.38	−0.10	0.06	0.25	0.12	0.65	−0.14	0.91	
UB	0.56	0.06	0.20	−0.07	0.23	0.43	0.05	0.33	0.36	−0.03	0.49	−0.05	F

^a^ *BI*: Behavioural intention, *CL*: Collection, *EE*: Effort expectancy, *ER*: Errors, *FC*: Facilitating conditions, *HT*: Habit, *HM*: Hedonic motivation, *PE*: Performance expectancy, *PV*: Price value, *SU*: Secondary use, *SI*: Social influence, *UA*: Unauthorized access, *UB*: Use behaviour, *F*: Formative

^b^ Diagonal elements are square roots of AVEs

^c^ Off-diagonal elements are correlations

**Table 6:** Correlations and square roots of AVEs in the US model

	BI	CL	EE	ER	FC	HT	HM	PE	PV	SU	SI	UA	UB
BI	0.94												
CL	−0.19	0.92											
EE	0.61	−0.18	0.94										
ER	0.23	−0.07	0.23	0.92									
FC	0.63	−0.20	0.79	0.33	0.83								
HT	0.46	0.01	0.29	−0.07	0.19	0.83							
HM	0.29	−0.08	0.31	−0.02	0.18	0.56	0.94						
PE	0.68	−0.25	0.55	0.31	0.62	0.37	0.35	0.95					
PV	0.58	−0.16	0.48	0.27	0.48	0.41	0.34	0.50	0.95				
SU	0.32	−0.12	0.36	0.55	0.51	−0.15	−0.08	0.34	0.31	0.88			
SI	0.45	−0.04	0.24	0.04	0.24	0.55	0.48	0.43	0.30	−0.04	0.96		
UA	0.33	−0.04	0.37	0.64	0.51	−0.11	−0.10	0.34	0.32	0.76	−0.07	0.91	
UB	0.62	−0.07	0.47	0.27	0.45	0.47	0.30	0.54	0.42	0.19	0.35	0.26	F

^a^ *BI*: Behavioural intention, *CL*: Collection, *EE*: Effort expectancy, *ER*: Errors, *FC*: Facilitating conditions, *HT*: Habit, *HM*: Hedonic motivation, *PE*: Performance expectancy, *PV*: Price value, *SU*: Secondary use, *SI*: Social influence, *UA*: Unauthorized access, *UB*: Use behaviour, *F*: Formative

^b^ Diagonal elements are square roots of AVEs

^c^ Off-diagonal elements are correlations

**Table 7:** Correlations and square roots of AVEs in the Portuguese model

	BI	CL	EE	ER	FC	HT	HM	PE	PV	SU	SI	UA	UB
BI	0.91												
CL	0.00	0.93											
EE	0.42	−0.17	0.89										
ER	0.05	−0.02	0.20	0.82									
FC	0.30	−0.07	0.56	0.17	0.79								
HT	0.63	0.14	0.29	−0.03	0.28	0.82							
HM	0.44	−0.02	0.45	0.07	0.34	0.50	0.94						
PE	0.50	0.03	0.46	0.19	0.27	0.44	0.48	0.91					
PV	0.36	0.00	0.35	0.05	0.27	0.47	0.44	0.33	0.86				
SU	−0.02	0.07	0.16	0.38	0.23	−0.09	0.08	0.12	−0.02	0.86			
SI	0.44	0.14	0.25	−0.07	0.25	0.57	0.32	0.32	0.34	−0.06	0.98		
UA	−0.11	−0.08	−0.23	−0.63	−0.25	−0.01	−0.13	−0.22	−0.08	−0.52	0.02	0.94	
UB	0.42	0.10	0.16	−0.06	0.22	0.41	0.16	0.22	0.21	−0.06	0.41	−0.04	F

^a^ *BI*: Behavioural intention, *CL*: Collection, *EE*: Effort expectancy, *ER*: Errors, *FC*: Facilitating conditions, *HT*: Habit, *HM*: Hedonic motivation, *PE*: Performance expectancy, *PV*: Price value, *SU*: Secondary use, *SI*: Social influence, *UA*: Unauthorized access, *UB*: Use behaviour, *F*: Formative

^b^ Diagonal elements are square roots of AVEs

^c^ Off-diagonal elements are correlations

**Table 8:** Formative indicators’ quality criteria

Indicators	VIF ^a^	T value (Weights) ^b^	Outer Loadings
Total			
UB1	3.41	3.64**	0.94
UB2	2.10	2.70**	0.81
UB3	3.17	3.65**	0.93
UB5	2.52	0.89	0.74
US			
UB1	2.45	23.41**	0.89
UB2	2.37	14.21**	0.79
UB3	1.98	25.59**	0.91
UB5	1.85	8.43**	0.61
Portugal			
UB1	2.72	16.72**	0.95
UB2	1.70	7.86**	0.81
UB3	3.26	7.74**	0.79
UB5	2.52	5.54**	0.68

^a^ *VIF*: Variance inflation factor; ^b^ ** = *P* < 0.01; * = *P* < 0.05. UB1 = Management of personal information and communication with health providers; UB2 = medical appointments schedule; UB3 = Check their own EHR; UB5 = Request for medical prescription renewals

In order to ensure good indicator reliability, an established rule of thumb is that the latent variable should explain more than half of the indicators’ variance [81]. The correlation between the constructs and their indicators should be equal to or higher than 0.7 ($\sqrt{0.5}$ ≈ 0.7) [75, 82]. Still, an item is definitively recommended to be eliminated only if its outer standardized loadings are lower than 0.4 [83]. The measurement model (total) that includes the full sample has issues with one indicator reliability, ER1, which was removed; FC4 and HT3 are lower than 0.7, but still higher than 0.4 (Additional file 2). Following the removal of ER1 both the Portuguese measurement model and the US measurement model had all of their outer standardized loadings higher than 0.4 (Additional file 2). We decided to keep the items with loadings between 0.4 and 0.7 in all three models (total, US, and Portugal) because their deletion in any of the models did not contributed to increase the average variance extracted (AVE) or CR above the suggested threshold values [81].

The most common measure to assess convergent validity in PLS-SEM is the AVE. Using the same basis as the one used with the individual indicators an AVE value of 50% or higher means that, on average, the construct explains more than half of the variance of its own indicators [81, 84]. As seen in Table 4, all of the indicators respect this criterion in all three models. Discriminant validity is the degree to which a construct is distinct from the other constructs in the model [84]. Two measures of discriminant validity can be used [81]. The first and more conservative is the Fornell- Larcker criterion [75, 84]. It states that the square root of each construct’s AVEs (diagonal elements) should be higher than its highest correlation with any other construct (off diagonal elements) [75, 84]. As seen in Tables 5, 6, and 7, this criterion is achieved in all three models. In addition, another criterion can be used to assess discriminant validity which is to examine the cross loadings of the indicators, although it is regarded as a more liberal one in terms of establishing discriminant validity [75]. Precisely in this criterion, an indicator loading on the associated construct should be higher than all of its loadings in the other constructs [76, 85]. This criterion is also met, as seen in Additional file 2.

Use, which was modelled using five formative indicators, is assessed by specific quality criteria related with formative indicators. In the total model collinearity issues were detected and UB4 (check your medical exam results) with variance inflation factor (VIF) of 6.03 was eliminated from the model. With the deletion of UB4 all remaining indicators, as seen in Table 8, are below 5, suggesting that multi-collinearity is not an issue [81]. Also the indicators’ weights comply with the criteria of being statistically significant, or in case they are not significant, its outer loading must be higher than 0.5 [81].

We also examined the common method variance (CMV) first using Harman's one-factor test. It revealed that the most variance explained by one factor, in this case the first factor, was 25.8%. None of the factors had variance more than the 50% threshold value [86]. Thereafter the marker-variable technique [87] was used, in which we employed a theoretically unrelated construct -the marker variable [87]. We found no significant correlation between the study constructs and the marker variable. We thus conclude that CMV was not a serious concern, tested by two different and known criteria [86–88].

Overall, all assessments are suitable. This means that the constructs may be used to test the conceptual model and its groups.

### Structural model

Structural model path significance levels were estimated using a bootstrap with 5000 iterations of resampling to acquire the maximum possible consistency in the results [81]. The R^2^ was used to assess the structural model. Overall the model explains 53% of the variance in behavioural intention and 36% of the variance in use behaviour. We used a modified version of the two-independent samples *t* test to compare path coefficients across two groups of data as described by Hair et al. [78] to perform PLS-SEM multi-group analysis (PLS-MGA). Behavioural intention R^2^ in the US group is higher than in the Portuguese group (64% versus 49%), use behaviour followed exactly the same trend (47% versus 23%). Table 9 presents the structural model results concerning the R^2^, path coefficients significance, and identifies the statistical significance difference between groups.

**Table 9:** Structural model results

Dependent variables	Independent variables	$\widehat{\beta}$_total_	$\widehat{\beta}$_PT_^a^	$\widehat{\beta}$_US_	*t*_*otal*_^*b*^	*t*_*PT*_^*b*^	*t*_*US*_^*b*^	$\widehat{\beta}$ *(*_*US –PT*_ *)*	*t (*_*US- PT*_ *)* ^*b*^	R^2^_Total_	R^2^_PT_	R^2^_US_
BI	PE	0.285	0.190	0.292	6.61**	3.29**	3.86**	0.102	1.07	0.53	0.49	0.64
	EE	0.160	0.177	0.163	3.17**	2.61**	1.99*	−0.014	0.13			
	SI	0.198	0.083	0.149	5.42**	1.57	2.91**	0.066	0.89			
	FC	0.062	0.001	0.181	1.51	0.02	2.15*	0.180	1.87			
	HM	−0.141	0.026	−0.138	3.63**	0.44	2.66**	−0.164	2.10*			
	PV	0.152	−0.004	0.196	3.62**	0.08	3.24**	0.200	2.46*			
	HT	0.255	0.436	0.188	6.74**	7.57**	3.60**	−0.248	3.20**			
UB	FC	0.052	0.103	0.106	1.19	2.09*	1.26	0.003	0.04	0.36	0.23	0.47
	HT	0.145	0.209	0.276	2.96**	2.59**	4.55**	0.067	0.67			
	CL	0.088	0.073	0.027	1.49	1.26	0.45	−0.046	0.56			
	ER	−0.018	−0.115	0.174	0.36	1.21	2.63**	0.289	2.50*			
	SU	0.000	−0.066	−0.091	0.00	0.76	1.16	−0.025	0.22			
	UA	−0.098	−0.085	0.064	1.47	0.76	0.70	0.149	1.03			
	BI	0.480	0.249	0.395	10.57**	3.30**	4.36**	0.146	1.26			

*PE*: Performance expectancy, *EE*: Effort expectancy, *SI*: Social influence, *FC*: Facilitating conditions, *HM*: Hedonic motivation, *PV*: Price value, HT Habit, BI Behavioural intention, *CL*: Collection, *ER*: Errors, *UA*: Unauthorized access, *SU*: Secondary use, *UB*: Use behaviour

^a^ *PT* Portugal; ^b^ ** = *P* < 0.01; * = *P* < 0.05

## Discussion

Our results seem to point out that in fact US and Portugal are in different stages, and that Portugal is still in the initial stage of adoption. Consequently, the factors determining user acceptance should differ in these two different stages [5, 17, 18]. The results reported in Table 3 seem to support these theoretical findings, suggesting that the Portuguese group is still in its initial stage of adoption with a low frequency of usage, whereas the US group seems to be already in the continued usage of EHR portals. Also, the factors that determine user acceptance are not exactly the same between the two groups. The more consistent and established use of EHR portals by the US group also seems to contribute to higher explanatory power of the model with the US sample versus the Portuguese sample [76, 77, 81]. The implementation of stage 2 meaningful use in the US leads to incentive payments to clinicians and hospitals [16], that according to recent reports have stimulated the adoption of EHR. These mandatory polices in the US, something that did not happen in Portugal to implement EHR portals [1], may have resulted in a greater effort to encourage the continuous usage of EHR portals by the patients when compared with Portugal.

### Theoretical implications

Performance expectancy ($\widehat{\beta}$_total_ = 0.285; *P* < 0.01) and effort expectancy ($\widehat{\beta}$_total_ = 0.160; *P* < 0.01) obtained statistically positive impacts on behaviour intention in the total model and in both groups, as reported in Table 9. Concerning the results obtained in studies that addressed similar issues, both performance and effort expectancy, originally from TAM [89], also had significant positive impacts, as reported in eHealth adoption studies including patient portals [20, 34]. These findings support both hypotheses H1 and H2, as reported in Table 10. Social influence ($\widehat{\beta}$_total_ = 0.198; *P* < 0.01) had a positive and significant impact on behaviour intention in the total model, supporting hypothesis H3, and also a statistically significant impact in the US group ($\widehat{\beta}$_US_ = 0.149; *P* < 0.01). Literature also supports that social influence could play a role in the adoption of eHealth platforms and that this influence may come from support groups and social media [40, 52]. Facilitating conditions did not show a significant impact in predicting behavioural intention and use behaviour in the total model. Although H4(a) and H4(b) are not supported in the total model, in the group analysis facilitating conditions ($\widehat{\beta}$_US_ = 0.181; *P* < 0.05) had a positive impact on behavioural intention in the US and a positive impact ($\widehat{\beta}$_PT_ = 0.103; *P* < 0.05) on use behaviour in Portugal. According to the literature, adoption and continued use of new IT technologies in general, but also in healthcare, represent different behavioural intention [5, 17, 18]. What the results seem to point out is that in a country like Portugal, in the initial stage of adoption, the availability of resources and support may directly increase use. Concerning the US, with an already higher frequency of usage, the availability of resources has a positive impact on behaviour intention, which promotes the continuous use of EHR Portals. Although there is some evidence in the literature to support these findings [17, 18], we believe that this topic should be further investigated in future studies, because when these results are analysed together the contributions of the non-significant paths of each country on the total model, result that their influence is to make H4 not significant (different facilitating conditions behaviours between the countries).

**Table 10:** Summary of findings regarding Hypotheses

Path	Beta	t-value	Hypothesis	Result
PE → BI	0.285	6.61**	H1	supported
EE → BI	0.160	3.17**	H2	supported
SI → BI	0.198	5.42**	H3	supported
FC → BI	0.062	1.51	H4(a)	not supported
FC → UB	0.052	1.19	H4(b)	not supported
HM → BI	−0.141	3.63**	H5	supported
PV → BI	0.152	3.62**	H6(a)	supported
(PV_US_ → BI_US_) - (PV_PT_ → BI_PT_)	0.200	2.46*	H6(b)	supported
HT → BI	0.255	6.74**	H7(a)	supported
HT → UB	0.145	2.96**	H7(b)	supported
BI→ UB	0.480	10.57**	H8	supported
CL→ UB	0.088	1.49	H9(a)	not supported
(CL_US_ → UB_US_) - (CL_PT_ → UB_PT_)	−0.046	0.56	H9(b)	not supported
ER→ UB	−0.018	0.36	H10(a)	not supported
(ER_US_ → UB_US_) - (ER_PT_ → UB_PT_)	0.289	2.50*	H10(b)	not supported
UA→ UB	−0.098	1.47	H11(a)	not supported
(UA_US_ → UB_US_) - (UA_PT_ → UB_PT_)	0.149	1.03	H11(b)	not supported
SU→ UB	0.000	0.00	H12(a)	not supported
(SU_US_ → UB_US_) - (SU_PT_ → UB_PT_)	−0.025	0.22	H12(b)	not supported

*PE*: Performance expectancy, *EE*: Effort expectancy, *SI*: Social influence, *FC*: Facilitating conditions, *HM*: Hedonic motivation, *PV*: Price value, *HT*: Habit, *BI*: Behavioural intention, *CL*: Collection, *ER*: Errors, *UA*: Unauthorized access, *SU*: Secondary use, *UB*: Use behaviour

** = *P* < 0.01; * = *P* < 0.05

We confirmed that hedonic motivation (H5) does have a significant negative effect ($\widehat{\beta}$_total_ = −0.141; *P* < 0.01) on behavioural intention. Another important finding is that the US group has a statistically significant difference versus the Portuguese group. In fact, this is the group that uses the EHR portals more frequently, and during its continuous usage does not perceive it as an enjoyment, but probably more as a need [3, 52]. Literature in healthcare shows that people using more health services and e-health have greater concerns about their health, more serious health problems, and have higher depression rates than the population average [34, 52–55]. Depression and poor health are also linked with less enjoyment in life [56, 57]. Literature points out that there are also other factors, different from the target’s hedonic proprieties, that influence engagement and can thus contribute to repulsion [53]. Therefore it is not surprising that patients do not regard the EHR Portal use as fun, because it is linked with a pre-existing health condition, and this is the factor different from the target’s hedonic proprieties, which contributes to the intensity of repulsion and the decrease of enjoyment [53]. This shows that findings from other consumer related areas that point out hedonic motivation as having a positive influence over adoption [19] do not necessarily apply in the case of EHR portals. Because in EHR Portals there is an external factor, different from the hedonic proprieties influencing the results, future research may use constructs related with the health belief model (HBM), such as perceived threat [35, 36], which links the perceived health concerns with the adoption of EHR Portals, which could be a more straightforward way to measure the same effect [35, 36].

Hypothesis H6(a), that price value ($\widehat{\beta}$_total_ = 0.152; *P* < 0.01) would have a positive impact on behavioural intention, was verified. There are also statistically significant differences between the US group and the Portuguese group, pointing out that in a healthcare context like the US’, where patients pay directly out of their pocket or via an expensive health insurance [5, 17], more value is attributed to the EHR portals’ added value of performing these activities in a more cost-effective manner, compared to the Portuguese patients, who are covered by an NHS that features universal coverage [13]. Our results, together with what is stated in the literature, support hypothesis H6(b), that patients with a PHI model coverage perceive greater price value advantages of an EHR portal than do patients with an NHS model [5, 13]. The construct habit has a statistically significant impact on both behavioural intention ($\widehat{\beta}$_total_ = 0.255; *P* < 0.01) and use behaviour ($\widehat{\beta}$_total_ = 0.145; *P* < 0.01), in line with findings from literature that refer habit as a predictor of behavioural intention and use behaviour in eHealth tools and EHR portals [3, 33], supporting both hypotheses H7(a) and H7(b). Our study’s findings are also in line with those of other studies, that using specific eHealth and EHR Portals is preceded by the intention to use ($\widehat{\beta}$_total_ = 0.480; *P* < 0.01) them [3, 35], supporting hypothesis H8.

**Fig. 2:**
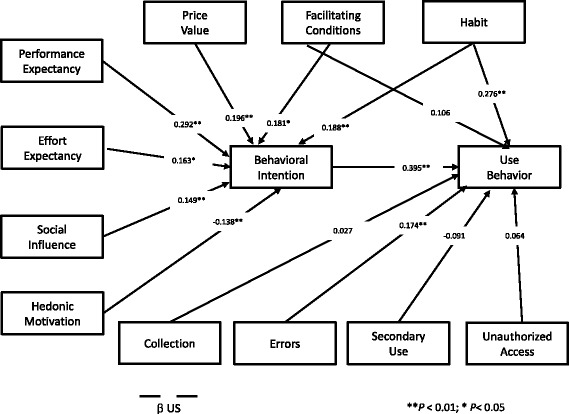
Structural model results for US

**Fig. 3:**
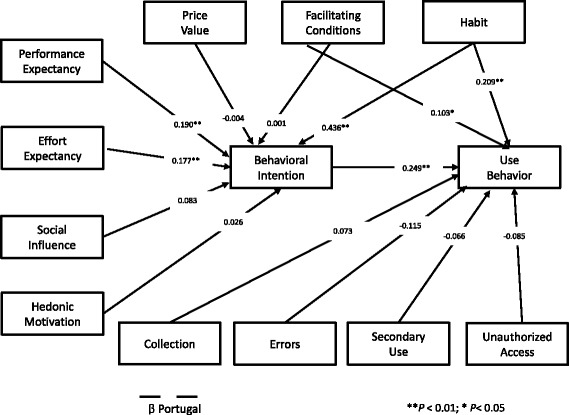
Structural model results for Portugal

The hypotheses related with CFIP constructs (H9-H12) were not supported. We tested people who know about the technology, adopt, and use it. People who already use EHR portals may have a different behaviour as compared with never users regarding confidentiality issues, and this may explain the unexpected behaviour toward confidentiality in our study [5]. One of the CFIP dimensions, Error ($\widehat{\beta}$_US_ = 0.174; *P* < 0.01), is linked in our study with a higher use of EHR portals in the US. This result may look surprising, but Angst and Agarwal [5] tested with success that individuals with a stronger Concern for Information Privacy should have a more favourable attitude toward EHR use under conditions of positive argumentation and communication in favour of EHR use. One possible explanation for this specific dimension from CFIP, and not the others, to be statistically significant is probably because the US patients perceive the reduction of medical errors as the biggest advantage of EHR [5], and they want to be reassured that the health entities comply with this objective. There is also a statistically significant difference between US and Portugal in the error dimension. Again, this is in line with the stage 2 meaningful use objective to promote the national use of EHR by the US patients versus Portugal, where this kind of national initiative was not implemented in a structured manner [1, 16]. This is a complex topic and its justification is far from being definitive. It only reinforces the literature findings that confidentiality issues in healthcare are a very complex topic [4, 5]. According to the literature, patient acceptance in consumer health technology is related to more educated and younger patients [17, 20]. The Portuguese sample is younger and more educated, but with less acceptance and usage. Nevertheless, both groups may be regarded as young, the US with an average of 36.42 years versus 30.80 of the Portuguese. Also regarding education, the Portuguese group has a greater proportion of people with more than a Bachelor’s degree. But in a more pragmatic approach, if we compare both groups with having or not a university degree, there are no statistically significant differences between the two groups. Overall the socio- demographics in our study do not seem to be relevant in the difference between the group’s results. Fig. 2 and Fig. 3 show the structural model results for each country.

### Managerial implications

A study that evaluates an important topic like EHR Portals should provide managerial insights that can be helpful in the design and implementation of this specific technology. That is exactly what we address in this section. Our study results point out that there is a significant impact of patients’ habit on EHR portals usage. Habit has been defined as the degree to which people tend to perform behaviours repeatedly because of learning [19]. So it is important that EHR Portals have customer support services to help users with the platform. Also, the fact that facilitating conditions seem to play a significant role (see Table 9) on use behaviour in the Portuguese group and behaviour intention in the US group is additional evidence in favour of customer service support, since the definition of facilitating conditions is related to perceptions of the resources and support available for a particular IT platform [19, 48]. The study also identified that both performance expectancy and effort expectancy have important influences on the adoption of EHR portals. Previous studies using TAM identified both constructs as being significant for the adoption of eHealth technologies and EHR portals, and suggest that these technologies should be simple and easy to use [3, 34, 37]. When redeploying or designing an EHR portal, we should thus strive to make it easy and simple for the healthcare consumers to use [21, 90, 91]. Social influence is also an important variable in the intention to use EHR portals, as demonstrated by the results of our study. Because this influence may come from online support groups, as reported in other studies [38, 52], digital strategies to promote eHealth tools by using social networks (e.g. Facebook) should be useful in promoting the adoption and use of EHR portals. Because price value is also a significant construct in our study, the value of the EHR portals and the way they may help patients to manage their health in a more cost-effective manner should be actively promoted to them. According to the literature, to avoid confidentiality concerns from reducing the acceptance of EHR portals, positive argumentation and communication in favour of their use should be actively promoted to patients [5]. There is evidence that a subset of patients during meaningful use, exposed to EHRs via their physicians, who explained the advantages of EHR, have more positive attitudes toward EHRs than those without that exposure [92].

### Limitations and future research

Only a small proportion of the population, less than 7%, uses EHR Portals [1–3, 69, 70], and according to the literature these individuals are younger and more educated than the population average [20, 73, 74]. This population profile is more concentrated in research and education institutions, making such places a good target for sampling, since this a suitable strategy to investigate low prevalence populations [71, 72]. Although the fact that our sampling is restricted to education and research institutions, and this can be regarded as a limitation of our study, it can be justified by the type of population we are targeting [71]. According to Karahanna et al. [18], adoption and continued use of an IT innovation represent different behavioural intentions. In our study, the US group is in a stage of continuous use of EHR Portals, unlike the situation in the Portuguese group. Taking these facts into account, Rogers’ innovation diffusion theory could be included in future models to study EHR Portals acceptance, as it was with other eHealth technologies [17]. Comparing people who already use EHR portals, as in our study, with those who never have in future studies (regarding confidentiality issues) may also explain different behaviour toward confidentiality [5]. Our study did not probed the EHR Portal users about the potential effect of positive message framing to which they may have been exposed, that could explain the non-impact of CFIP on adoption [5, 92], and future studies may address this topic. Constructs related with the HBM such as perceived threat may replace Hedonic Motivation in future studies, since they provide a more direct measure of the intrinsic motivation of the patients toward EHR Portals [35, 36]. The CFIP framework did not reveal a statistically significant role in our study, but provided theoretical and managerial insights that invite further analysis in future studies. PLS path modelling is primarily used to develop theories in exploratory research [81]. It does this by focusing on explaining the variance in the dependent variables when examining the model and is particularly suitable for multi-group analysis [76, 81, 93] aligned with our study goals. PLS-SEM does not have an adequate global goodness-of-model fit measure, and its use for confirmatory theory testing is limited, and in this case covariance based (CB)-SEM is a more appropriate option [81, 93], and should be used in future studies when more information about the study context is gathered, and other constructs, moderators, or theories beyond CFIP could play a more significant role.

## Conclusions

EHR portals adoption is a recent and emergent field of study that is an important topic in both the EU and the US [1, 16]. Among the constructs tested, performance expectancy, effort expectancy, social influence, hedonic motivation (negative influence), price value, and habit had the most significant effects over behavioural intention. Habit and behavioural intention had a significant effect over use behaviour. Price value had a statistically significant impact on behaviour intention in the US group in opposition to the non-significant impact of the Portuguese group. Also regarding price value, the differences between groups are significant, demonstrating that in a country like the US, where the healthcare cost is very expensive to the patient, the value of EHR portals is better perceived by the patients [1, 5, 13]. Our study focused on healthcare consumers who are already users of EHR Portals, and found that confidentiality concerns do not decrease the current usage of EHR Portals by the patients or healthcare consumers. Other studies that focused on the intention to use [46], report that confidentiality concerns could be a barrier for future use. It seems that when someone starts using an EHR Portal, the impact of confidentiality concerns on effective use is not significant. It seems that when a patient overcomes the barrier of potential intention to use, to effective opt-in use of an EHR Portal, confidentiality concerns, measured via CFIP in our study are no longer a significant obstacle. There is evidence in the literature that with positive argumentation about EHR Portals, confidentiality concerns will no longer significantly impact adoption [5]. There is recent literature about the on-going implementation of meaningful use that seems to support this evidence [92]. In any event our study is exploring a very recent topic, studying effective users of EHR Portals and future studies are required to evaluate our study findings even deeper. Overall, the model explains 53% and 36% of the variance in behavioural intention and use behaviour, with these values being higher in the US group, 64% on behaviour intention and 47% on use behaviour. The US group also reveals much higher and significant usage patterns compared with the Portuguese group. We applied the results obtained in this research to deliver managerial insights that may increase the usage and adoption of EHR portals.

## Additional files

Additional file 1: Questionnaire items.

Additional file 2: PLS loadings and cross-loadings (includes the data from the three models).

## Abbreviations

CFIP: Concern for Information Privacy
CMS: Center for Medicare & Medicaid Services
EHR: Electronic Health Record
PLS: Partial least squares
TAM: Technology Acceptance Model
UTAUT: Unified Theory of Acceptance and Use of Technology
UTAUT2: Extended Unified Theory of Acceptance and Use of Technology in a consumer context
