# Supplementary material for: Electronic Health Record Portal Adoption: a cross country analysis
Source: BMC Med Inform Decis Mak. 2017 Jul 5;17:97. doi: 10.1186/s12911-017-0482-9 (PMC5499062; doi:10.1186/s12911-017-0482-9)
Supplement: Supplementary file 1 — Questionnaire items. [file 12911_2017_482_MOESM1_ESM.docx]

## Additional file 1. Questionnaire items

| The scales’ items were measured on a seven-point Likert scale, ranging from “strongly disagree” (1) to “strongly agree” (7). Use was measured on a different scale (explained in the table below).   \| Construct \| Code \| Items \| Reference \| \| --- \| --- \| --- \| --- \| \| Performance Expectancy \| PE1 \| Using EHR Portals will support critical aspects of my healthcare. \| [34] \| \| PE2 \| Using EHR Portals will enhance my effectiveness in managing my healthcare. \| \| PE3 \| Overall, EHR Portals will be useful in managing my healthcare. \| \| Effort Expectancy \| EE1 \| Learning how to use EHR Portals is easy for me. \| [19] \| \| EE2 \| My interaction with EHR Portals is clear and understandable. \| \| EE3 \| I find EHR Portals easy to use. \| \| EE4 \| It is easy for me to become skilful at using EHR Portals. \| \| Social Influence \| SI1 \| People who are important to me think that I should use EHR Portals. \| [19] \| \| SI2 \| People who influence my behaviour think that I should use EHR Portals. \| \| SI3 \| People whose opinions that I value prefer that I use EHR Portals. \| \| Facilitating Conditions \| FC1 \| I have the resources necessary to use EHR Portals. \| [19] \| \| FC2 \| I have the knowledge necessary to use EHR Portals. \| \| FC3 \| EHR Portals are compatible with other technologies I use. \| \| FC4 \| I can get help from others when I have difficulties using EHR Portals. \| \| Hedonic Motivation \| HM1 \| Using EHR Portals is fun. \| [19] \| \| HM2 \| Using EHR Portals is enjoyable. \| \| HM3 \| Using EHR Portals is very entertaining. \| \| Price Value \| PV1 \| EHR Portals is reasonably priced. \| [19] \| \| PV2 \| EHR Portals is a good value for the money. \| \| PV3 \| At the current price, EHR Portals provides a good value. \| \| Habit \| HT1 \| The use of EHR Portals has become a habit for me. \| [19] \| \| HT2 \| I am addicted to using EHR Portals. \| \| HT3 \| I must use EHR Portals. \| \| Collection \| CL1 \| It usually bothers me when healthcare entities ask me for personal information. \| [5] \| \| CL2 \| When healthcare entities ask me for personal information, I sometimes think twice before providing it. \| \| CL3 \| It bothers me to give personal information to so many healthcare entities. \| \| CL4 \| I’m concerned that healthcare entities are collecting too much personal information about me. \| \| Errors \| ER1 \| All the personal information in computer databases should be double-checked for accuracy—no matter how much this costs. (dropped) \| [5] \| \| ER2 \| Healthcare entities should take more steps to make sure that the personal information in their files is accurate. \| \| ER3 \| Healthcare entities should have better procedures to correct errors in personal information. \| \| ER4 \| Healthcare entities should devote more time and effort to verifying the accuracy of the personal information in their databases. \| \| Secondary Use \| SU1 \| Healthcare entities should not use personal information for any purpose unless it has been authorized by the individuals who provided the information. \| [5] \| \| SU2 \| When people give personal information to a company for some reason, the company should never use the information for any other reason. \| \| SU3 \| Healthcare entities should never sell the personal information in their computer databases to other healthcare entities. \| \| SU4 \| Healthcare entities should never share personal information with other healthcare entities unless it has been authorized by the patient who provided the information \| \| Unauthorized Access \| UA1 \| Healthcare entities should devote more time and effort to preventing unauthorized access to personal information. \| [5] \| \| UA2 \| Computer databases that contain personal information should be protected from unauthorized access no matter how much it costs. \| \| UA3 \| Healthcare entities should take more steps to make sure that unauthorized people cannot access personal information in their computers. \| \| Behavioural Intention \| BI1 \| I intend to use EHR Portals. \| [19] \| \| BI2 \| I intend to use EHR Portals in the next months. \| \| BI3 \| I plan to use EHR Portals frequently. \| \| Use Behaviour \|  \| What is your actual frequency of use of the following EHR Portal services? (1) Never; to (7) every time I need it. \| [19] \| \| UB1 \| Management of Personal Information and communication with health providers. \| \| UB2 \| Medical appointments schedule. \| \| UB3 \| Check your own Electronic Health Record. \| \| UB4 \| Check your medical exams results (dropped) \| \|  \| UB5 \| Request for medical prescription renewals. \|  \| |
| --- | --- | --- | --- | --- | --- | --- | --- | --- | --- | --- | --- | --- | --- | --- | --- | --- | --- | --- | --- | --- | --- | --- | --- | --- | --- | --- | --- | --- | --- | --- | --- | --- | --- | --- | --- | --- | --- | --- | --- | --- | --- | --- | --- | --- | --- | --- | --- | --- | --- | --- | --- | --- | --- | --- | --- | --- | --- | --- | --- | --- | --- | --- | --- | --- | --- | --- | --- | --- | --- | --- | --- | --- | --- | --- | --- | --- | --- | --- | --- | --- | --- | --- | --- | --- | --- | --- | --- | --- | --- | --- | --- | --- | --- | --- | --- | --- | --- | --- | --- | --- | --- | --- | --- | --- | --- | --- | --- | --- | --- | --- | --- | --- | --- | --- | --- | --- | --- | --- | --- | --- | --- | --- | --- | --- | --- | --- |

**Introduction about EHR Portals presented to respondents before administering the questionnaire:**

Electronic health record portals are based on applying information technologies and systems on health environments. These portals allow, for instance, to make medical appointments online, to access medical history, medication records, specialists’ summaries, and laboratory results. The access to these services is made through a web page, and allows you, as a patient, to manage your medical records. Please answer the questionnaire only if you have prior knowledge and contact with electronic health record portals. When we mention “EHR Portals” in this questionnaire, it refers to electronic health record portals.
