# Supplementary material for: Electronic Health Record Portal Adoption: a cross country analysis
Source: BMC Med Inform Decis Mak. 2017 Jul 5;17:97. doi: 10.1186/s12911-017-0482-9 (PMC5499062; doi:10.1186/s12911-017-0482-9)
Supplement: Supplementary file 2 — PLS loadings and cross-loadings (includes the data from the three models). [file 12911_2017_482_MOESM2_ESM.docx]

## Additional file 2- PLS loadings and cross-loadings

Table A1- PLS loadings and cross-loadings total model

| Construct | Item | BI | CL | ER | SU | UA | EE | FC | HT | HM | PE | PV | SI |
| --- | --- | --- | --- | --- | --- | --- | --- | --- | --- | --- | --- | --- | --- |
| Behavioural intention | BI1 | **0.91** | -0.10 | 0.12 | 0.15 | 0.17 | 0.50 | 0.44 | 0.50 | 0.32 | 0.60 | 0.44 | 0.43 |
|  | BI2 | **0.93** | -0.01 | 0.01 | 0.05 | 0.08 | 0.40 | 0.34 | 0.52 | 0.25 | 0.50 | 0.45 | 0.47 |
|  | BI3 | **0.92** | -0.05 | -0.01 | 0.04 | 0.04 | 0.36 | 0.34 | 0.46 | 0.18 | 0.48 | 0.47 | 0.50 |
| Collection | CL1 | -0.10 | **0.71** | -0.04 | -0.04 | 0.01 | -0.16 | -0.14 | 0.10 | -0.04 | -0.12 | -0.11 | 0.03 |
|  | CL2 | -0.07 | **0.87** | -0.04 | -0.06 | -0.01 | -0.16 | -0.13 | 0.08 | -0.02 | -0.11 | -0.04 | 0.06 |
|  | CL3 | -0.07 | **0.86** | 0.04 | 0.03 | 0.07 | -0.13 | -0.08 | 0.04 | -0.05 | -0.06 | -0.06 | 0.03 |
|  | CL4 | -0.07 | **0.97** | -0.01 | -0.03 | 0.03 | -0.18 | -0.12 | 0.08 | -0.05 | -0.09 | -0.05 | 0.07 |
| Errors | ER2 | 0.08 | 0.00 | **0.93** | 0.51 | 0.68 | 0.24 | 0.29 | -0.07 | 0.09 | 0.25 | 0.13 | -0.07 |
|  | ER3 | 0.03 | -0.01 | **0.99** | 0.49 | 0.66 | 0.21 | 0.24 | -0.11 | 0.07 | 0.23 | 0.07 | -0.12 |
|  | ER4 | 0.03 | 0.08 | **0.85** | 0.43 | 0.60 | 0.17 | 0.21 | -0.03 | 0.10 | 0.21 | 0.10 | -0.06 |
| Secondary use | SU1 | 0.09 | -0.03 | 0.48 | **0.91** | 0.61 | 0.27 | 0.36 | -0.14 | 0.03 | 0.22 | 0.10 | -0.11 |
|  | SU2 | 0.07 | -0.01 | 0.46 | **0.90** | 0.58 | 0.27 | 0.32 | -0.09 | 0.04 | 0.18 | 0.10 | -0.10 |
|  | SU3 | 0.07 | 0.01 | 0.42 | **0.75** | 0.52 | 0.22 | 0.30 | -0.17 | 0.02 | 0.19 | 0.08 | -0.08 |
|  | SU4 | 0.08 | -0.05 | 0.39 | **0.79** | 0.50 | 0.19 | 0.29 | -0.14 | 0.01 | 0.17 | 0.09 | -0.03 |
| Unauthorized access | UA1 | 0.11 | 0.05 | 0.65 | 0.61 | **0.95** | 0.30 | 0.36 | -0.09 | 0.06 | 0.24 | 0.11 | -0.13 |
|  | UA2 | 0.14 | 0.02 | 0.61 | 0.60 | **0.82** | 0.29 | 0.35 | -0.07 | 0.05 | 0.25 | 0.16 | -0.09 |
|  | UA3 | 0.09 | 0.01 | 0.67 | 0.63 | **0.96** | 0.30 | 0.37 | -0.10 | 0.06 | 0.23 | 0.12 | -0.15 |
| Effort expectancy | EE1 | 0.37 | -0.17 | 0.24 | 0.27 | 0.33 | **0.90** | 0.63 | 0.17 | 0.32 | 0.40 | 0.30 | 0.08 |
|  | EE2 | 0.44 | -0.15 | 0.17 | 0.22 | 0.24 | **0.93** | 0.60 | 0.28 | 0.38 | 0.48 | 0.39 | 0.24 |
|  | EE3 | 0.41 | -0.14 | 0.17 | 0.24 | 0.26 | **0.90** | 0.61 | 0.27 | 0.36 | 0.46 | 0.38 | 0.22 |
|  | EE4 | 0.42 | -0.16 | 0.26 | 0.30 | 0.32 | **0.92** | 0.64 | 0.23 | 0.34 | 0.47 | 0.35 | 0.16 |
| Facilitating conditions | FC1 | 0.30 | -0.12 | 0.25 | 0.37 | 0.36 | 0.55 | **0.82** | 0.13 | 0.15 | 0.33 | 0.25 | 0.07 |
|  | FC2 | 0.37 | -0.08 | 0.26 | 0.35 | 0.38 | 0.62 | **0.90** | 0.20 | 0.21 | 0.38 | 0.33 | 0.17 |
|  | FC3 | 0.37 | -0.12 | 0.25 | 0.30 | 0.32 | 0.62 | **0.84** | 0.15 | 0.22 | 0.40 | 0.25 | 0.12 |
|  | FC4 | 0.25 | -0.04 | 0.06 | 0.14 | 0.15 | 0.37 | **0.64** | 0.25 | 0.30 | 0.23 | 0.29 | 0.31 |
| Habit | HT1 | 0.59 | 0.03 | -0.09 | -0.08 | -0.06 | 0.29 | 0.28 | **0.93** | 0.34 | 0.40 | 0.47 | 0.59 |
|  | HT2 | 0.26 | 0.13 | -0.16 | -0.23 | -0.22 | 0.05 | 0.03 | **0.78** | 0.44 | 0.15 | 0.26 | 0.42 |
|  | HT3 | 0.27 | 0.04 | 0.07 | -0.02 | 0.02 | 0.23 | 0.13 | **0.62** | 0.50 | 0.31 | 0.21 | 0.21 |
| Hedonic motivation | HM1 | 0.28 | -0.04 | 0.05 | 0.02 | 0.06 | 0.34 | 0.25 | 0.45 | **0.95** | 0.38 | 0.34 | 0.28 |
|  | HM2 | 0.27 | -0.05 | 0.11 | 0.07 | 0.10 | 0.43 | 0.31 | 0.43 | **0.93** | 0.41 | 0.31 | 0.25 |
|  | HM3 | 0.21 | -0.03 | 0.06 | 0.00 | 0.02 | 0.30 | 0.19 | 0.43 | **0.94** | 0.30 | 0.27 | 0.24 |
| Performance expectancy | PE1 | 0.41 | -0.10 | 0.27 | 0.22 | 0.27 | 0.43 | 0.36 | 0.30 | 0.38 | **0.87** | 0.31 | 0.24 |
|  | PE2 | 0.56 | -0.06 | 0.23 | 0.21 | 0.23 | 0.48 | 0.42 | 0.35 | 0.36 | **0.95** | 0.39 | 0.34 |
|  | PE3 | 0.57 | -0.06 | 0.19 | 0.18 | 0.19 | 0.46 | 0.39 | 0.39 | 0.34 | **0.94** | 0.42 | 0.40 |
| Price value | PV1 | 0.43 | 0.02 | 0.10 | 0.13 | 0.13 | 0.34 | 0.32 | 0.38 | 0.26 | 0.32 | **0.92** | 0.30 |
|  | PV2 | 0.48 | -0.04 | 0.09 | 0.12 | 0.12 | 0.39 | 0.35 | 0.43 | 0.34 | 0.43 | **0.96** | 0.36 |
|  | PV3 | 0.49 | -0.05 | 0.08 | 0.07 | 0.10 | 0.38 | 0.33 | 0.44 | 0.33 | 0.41 | **0.96** | 0.38 |
| Social influence | SI1 | 0.49 | 0.07 | -0.10 | -0.09 | -0.13 | 0.19 | 0.20 | 0.56 | 0.25 | 0.36 | 0.37 | **0.97** |
|  | SI2 | 0.49 | 0.08 | -0.11 | -0.10 | -0.14 | 0.20 | 0.20 | 0.55 | 0.28 | 0.34 | 0.35 | **0.98** |
|  | SI3 | 0.50 | 0.07 | -0.12 | -0.10 | -0.14 | 0.18 | 0.20 | 0.57 | 0.28 | 0.36 | 0.36 | **0.98** |

Table A2- PLS loadings and cross-loadings US model

| Construct | Item | BI | CL | ER | SU | UA | EE | FC | HT | HM | PE | PV | SI |
| --- | --- | --- | --- | --- | --- | --- | --- | --- | --- | --- | --- | --- | --- |
| Behavioural intention | BI1 | **0.96** | -0.16 | 0.22 | 0.31 | 0.33 | 0.60 | 0.62 | 0.44 | 0.28 | 0.67 | 0.54 | 0.43 |
|  | BI2 | **0.92** | -0.14 | 0.13 | 0.22 | 0.24 | 0.51 | 0.53 | 0.51 | 0.27 | 0.58 | 0.56 | 0.45 |
|  | BI3 | **0.95** | -0.23 | 0.29 | 0.36 | 0.37 | 0.61 | 0.63 | 0.36 | 0.27 | 0.66 | 0.55 | 0.40 |
| Collection | CL1 | -0.20 | **0.92** | -0.13 | -0.16 | -0.09 | -0.17 | -0.20 | 0.08 | -0.05 | -0.28 | -0.16 | -0.02 |
|  | CL2 | -0.20 | **0.93** | -0.11 | -0.16 | -0.10 | -0.21 | -0.24 | 0.00 | -0.06 | -0.26 | -0.13 | -0.02 |
|  | CL3 | -0.13 | **0.94** | 0.00 | -0.02 | 0.04 | -0.11 | -0.12 | -0.04 | -0.09 | -0.17 | -0.12 | -0.06 |
|  | CL4 | -0.19 | **0.91** | -0.03 | -0.12 | 0.00 | -0.19 | -0.20 | -0.01 | -0.09 | -0.25 | -0.17 | -0.04 |
| Errors | ER2 | 0.25 | -0.07 | **0.92** | 0.57 | 0.64 | 0.25 | 0.36 | -0.06 | -0.01 | 0.33 | 0.28 | 0.06 |
|  | ER3 | 0.21 | -0.09 | **0.91** | 0.52 | 0.60 | 0.21 | 0.31 | -0.12 | -0.05 | 0.28 | 0.22 | 0.00 |
|  | ER4 | 0.18 | -0.04 | **0.93** | 0.43 | 0.55 | 0.18 | 0.25 | -0.01 | 0.01 | 0.25 | 0.24 | 0.04 |
| Secondary use | SU1 | 0.32 | -0.16 | 0.50 | **0.91** | 0.72 | 0.35 | 0.50 | -0.13 | -0.10 | 0.34 | 0.29 | -0.03 |
|  | SU2 | 0.28 | -0.08 | 0.51 | **0.88** | 0.68 | 0.31 | 0.40 | -0.08 | -0.07 | 0.28 | 0.25 | -0.05 |
|  | SU3 | 0.27 | -0.10 | 0.46 | **0.86** | 0.61 | 0.31 | 0.45 | -0.18 | -0.04 | 0.31 | 0.29 | -0.02 |
|  | SU4 | 0.24 | -0.10 | 0.44 | **0.87** | 0.66 | 0.30 | 0.45 | -0.17 | -0.06 | 0.29 | 0.26 | -0.04 |
| Unauthorized access | UA1 | 0.32 | 0.00 | 0.58 | 0.65 | **0.90** | 0.32 | 0.44 | -0.11 | -0.07 | 0.32 | 0.25 | -0.07 |
|  | UA2 | 0.31 | -0.07 | 0.54 | 0.72 | **0.90** | 0.35 | 0.48 | -0.09 | -0.10 | 0.32 | 0.34 | -0.03 |
|  | UA3 | 0.27 | -0.03 | 0.64 | 0.71 | **0.91** | 0.33 | 0.47 | -0.11 | -0.09 | 0.30 | 0.28 | -0.09 |
| Effort expectancy | EE1 | 0.56 | -0.16 | 0.22 | 0.35 | 0.37 | **0.94** | 0.74 | 0.24 | 0.26 | 0.48 | 0.43 | 0.17 |
|  | EE2 | 0.56 | -0.15 | 0.17 | 0.28 | 0.30 | **0.93** | 0.71 | 0.32 | 0.33 | 0.51 | 0.46 | 0.26 |
|  | EE3 | 0.57 | -0.17 | 0.20 | 0.33 | 0.31 | **0.93** | 0.74 | 0.28 | 0.29 | 0.53 | 0.43 | 0.25 |
|  | EE4 | 0.58 | -0.18 | 0.26 | 0.39 | 0.39 | **0.94** | 0.78 | 0.25 | 0.28 | 0.53 | 0.48 | 0.22 |
| Facilitating conditions | FC1 | 0.54 | -0.18 | 0.30 | 0.49 | 0.48 | 0.67 | **0.86** | 0.12 | 0.11 | 0.50 | 0.43 | 0.13 |
|  | FC2 | 0.58 | -0.17 | 0.30 | 0.49 | 0.48 | 0.74 | **0.90** | 0.20 | 0.16 | 0.57 | 0.47 | 0.20 |
|  | FC3 | 0.59 | -0.16 | 0.33 | 0.44 | 0.44 | 0.71 | **0.89** | 0.13 | 0.12 | 0.56 | 0.39 | 0.17 |
|  | FC4 | 0.36 | -0.15 | 0.12 | 0.22 | 0.25 | 0.49 | **0.66** | 0.20 | 0.25 | 0.41 | 0.29 | 0.34 |
| Habit | HT1 | 0.58 | -0.07 | 0.00 | -0.02 | 0.03 | 0.40 | 0.34 | **0.93** | 0.49 | 0.48 | 0.47 | 0.50 |
|  | HT2 | 0.08 | 0.15 | -0.17 | -0.35 | -0.33 | -0.02 | -0.14 | **0.72** | 0.51 | 0.05 | 0.12 | 0.43 |
|  | HT3 | 0.23 | 0.07 | -0.12 | -0.23 | -0.22 | 0.10 | -0.02 | **0.84** | 0.46 | 0.16 | 0.25 | 0.45 |
| Hedonic motivation | HM1 | 0.27 | -0.07 | -0.02 | -0.06 | -0.06 | 0.29 | 0.18 | 0.51 | **0.95** | 0.34 | 0.32 | 0.44 |
|  | HM2 | 0.32 | -0.10 | 0.00 | -0.05 | -0.08 | 0.34 | 0.22 | 0.55 | **0.96** | 0.38 | 0.37 | 0.47 |
|  | HM3 | 0.17 | -0.01 | -0.04 | -0.15 | -0.17 | 0.20 | 0.06 | 0.50 | **0.90** | 0.21 | 0.22 | 0.42 |
| Performance expectancy | PE1 | 0.61 | -0.26 | 0.27 | 0.29 | 0.28 | 0.47 | 0.52 | 0.39 | 0.33 | **0.93** | 0.44 | 0.43 |
|  | PE2 | 0.65 | -0.23 | 0.31 | 0.33 | 0.33 | 0.54 | 0.62 | 0.33 | 0.30 | **0.95** | 0.49 | 0.39 |
|  | PE3 | 0.66 | -0.23 | 0.30 | 0.34 | 0.36 | 0.54 | 0.61 | 0.34 | 0.34 | **0.96** | 0.50 | 0.41 |
| Price value | PV1 | 0.50 | -0.08 | 0.28 | 0.33 | 0.32 | 0.44 | 0.45 | 0.36 | 0.25 | 0.42 | **0.93** | 0.22 |
|  | PV2 | 0.59 | -0.19 | 0.24 | 0.29 | 0.31 | 0.47 | 0.47 | 0.41 | 0.35 | 0.51 | **0.96** | 0.32 |
|  | PV3 | 0.57 | -0.17 | 0.25 | 0.26 | 0.30 | 0.46 | 0.46 | 0.39 | 0.36 | 0.50 | **0.96** | 0.32 |
| Social influence | SI1 | 0.44 | -0.04 | 0.04 | -0.04 | -0.05 | 0.24 | 0.24 | 0.52 | 0.47 | 0.42 | 0.32 | **0.96** |
|  | SI2 | 0.42 | -0.03 | 0.02 | -0.04 | -0.08 | 0.25 | 0.23 | 0.52 | 0.46 | 0.39 | 0.27 | **0.96** |
|  | SI3 | 0.44 | -0.03 | 0.04 | -0.03 | -0.07 | 0.21 | 0.22 | 0.54 | 0.45 | 0.43 | 0.28 | **0.97** |

Table A3- PLS loadings and cross-loadings Portugal model

| Construct | Item | BI | CL | ER | SU | UA | EE | FC | HT | HM | PE | PV | SI |
| --- | --- | --- | --- | --- | --- | --- | --- | --- | --- | --- | --- | --- | --- |
| Behavioural intention | BI1 | **0.89** | -0.06 | 0.11 | 0.04 | -0.15 | 0.47 | 0.33 | 0.59 | 0.46 | 0.53 | 0.33 | 0.39 |
|  | BI2 | **0.93** | 0.05 | 0.06 | -0.01 | -0.11 | 0.36 | 0.24 | 0.55 | 0.38 | 0.43 | 0.32 | 0.40 |
|  | BI3 | **0.90** | 0.03 | -0.03 | -0.09 | -0.03 | 0.31 | 0.24 | 0.58 | 0.36 | 0.40 | 0.35 | 0.42 |
| Collection | CL1 | 0.02 | **0.91** | -0.04 | 0.07 | -0.06 | -0.18 | -0.10 | 0.12 | -0.05 | 0.02 | -0.05 | 0.12 |
|  | CL2 | 0.02 | **0.95** | -0.02 | 0.04 | -0.09 | -0.12 | -0.05 | 0.15 | 0.00 | 0.02 | 0.03 | 0.13 |
|  | CL3 | -0.03 | **0.94** | 0.02 | 0.09 | -0.07 | -0.16 | -0.06 | 0.11 | -0.03 | 0.04 | -0.01 | 0.11 |
|  | CL4 | 0.00 | **0.92** | -0.04 | 0.07 | -0.08 | -0.17 | -0.05 | 0.14 | 0.00 | 0.05 | 0.03 | 0.15 |
| Errors | ER2 | 0.06 | 0.05 | **0.92** | 0.42 | -0.70 | 0.20 | 0.20 | 0.01 | 0.10 | 0.21 | 0.08 | -0.04 |
|  | ER3 | 0.05 | 0.04 | **0.92** | 0.43 | -0.68 | 0.18 | 0.16 | -0.01 | 0.08 | 0.22 | 0.05 | -0.05 |
|  | ER4 | 0.04 | 0.14 | **0.59** | 0.39 | -0.62 | 0.13 | 0.16 | 0.03 | 0.10 | 0.20 | 0.07 | 0.00 |
| Secondary use | SU1 | -0.02 | 0.07 | 0.40 | **0.84** | -0.52 | 0.15 | 0.22 | -0.08 | 0.11 | 0.14 | -0.01 | -0.09 |
|  | SU2 | -0.02 | 0.06 | 0.34 | **0.89** | -0.45 | 0.20 | 0.22 | -0.04 | 0.09 | 0.11 | 0.02 | -0.05 |
|  | SU3 | -0.04 | 0.12 | 0.34 | **0.88** | -0.46 | 0.12 | 0.17 | -0.12 | 0.05 | 0.09 | -0.07 | -0.10 |
|  | SU4 | 0.00 | 0.03 | 0.26 | **0.82** | -0.39 | 0.08 | 0.16 | -0.09 | 0.04 | 0.09 | -0.02 | 0.00 |
| Unauthorized access | UA1 | 0.09 | 0.09 | 0.62 | 0.52 | **-0.91** | 0.24 | 0.26 | 0.02 | 0.10 | 0.20 | 0.08 | -0.01 |
|  | UA2 | 0.12 | 0.10 | 0.55 | 0.44 | **-0.94** | 0.18 | 0.21 | 0.02 | 0.12 | 0.22 | 0.07 | -0.01 |
|  | UA3 | 0.10 | 0.05 | 0.62 | 0.52 | **-0.98** | 0.22 | 0.26 | -0.01 | 0.13 | 0.21 | 0.08 | -0.03 |
| Effort expectancy | EE1 | 0.36 | -0.19 | 0.16 | 0.13 | -0.21 | **0.87** | 0.52 | 0.19 | 0.33 | 0.35 | 0.27 | 0.15 |
|  | EE2 | 0.40 | -0.15 | 0.18 | 0.15 | -0.18 | **0.91** | 0.49 | 0.28 | 0.46 | 0.47 | 0.34 | 0.27 |
|  | EE3 | 0.33 | -0.11 | 0.15 | 0.13 | -0.19 | **0.86** | 0.49 | 0.29 | 0.46 | 0.39 | 0.36 | 0.24 |
|  | EE4 | 0.40 | -0.14 | 0.22 | 0.17 | -0.22 | **0.91** | 0.51 | 0.27 | 0.38 | 0.43 | 0.29 | 0.21 |
| Facilitating conditions | FC1 | 0.20 | -0.07 | 0.14 | 0.22 | -0.22 | 0.41 | **0.79** | 0.17 | 0.17 | 0.17 | 0.14 | 0.11 |
|  | FC2 | 0.27 | -0.02 | 0.19 | 0.23 | -0.29 | 0.49 | **0.90** | 0.23 | 0.24 | 0.24 | 0.25 | 0.20 |
|  | FC3 | 0.28 | -0.12 | 0.13 | 0.17 | -0.17 | 0.53 | **0.82** | 0.20 | 0.29 | 0.28 | 0.18 | 0.17 |
|  | FC4 | 0.17 | 0.01 | 0.05 | 0.11 | -0.10 | 0.31 | **0.61** | 0.29 | 0.36 | 0.12 | 0.29 | 0.31 |
| Habit | HT1 | 0.54 | 0.12 | -0.07 | -0.10 | 0.02 | 0.23 | 0.26 | **0.88** | 0.34 | 0.32 | 0.43 | 0.61 |
|  | HT2 | 0.41 | 0.14 | -0.12 | -0.14 | 0.06 | 0.13 | 0.18 | **0.81** | 0.41 | 0.26 | 0.37 | 0.44 |
|  | HT3 | 0.57 | 0.08 | 0.12 | 0.02 | -0.09 | 0.33 | 0.23 | **0.76** | 0.48 | 0.50 | 0.34 | 0.32 |
| Hedonic motivation | HM1 | 0.41 | -0.03 | 0.03 | 0.05 | -0.11 | 0.38 | 0.29 | 0.48 | **0.95** | 0.43 | 0.43 | 0.31 |
|  | HM2 | 0.42 | -0.01 | 0.11 | 0.12 | -0.15 | 0.51 | 0.39 | 0.45 | **0.90** | 0.50 | 0.38 | 0.29 |
|  | HM3 | 0.41 | -0.02 | 0.05 | 0.06 | -0.09 | 0.39 | 0.27 | 0.47 | **0.95** | 0.41 | 0.42 | 0.30 |
| Performance expectancy | PE1 | 0.37 | 0.02 | 0.22 | 0.12 | -0.23 | 0.39 | 0.20 | 0.31 | 0.40 | **0.86** | 0.27 | 0.22 |
|  | PE2 | 0.50 | 0.06 | 0.16 | 0.13 | -0.22 | 0.44 | 0.26 | 0.42 | 0.46 | **0.95** | 0.30 | 0.30 |
|  | PE3 | 0.48 | 0.01 | 0.16 | 0.09 | -0.17 | 0.44 | 0.25 | 0.46 | 0.43 | **0.92** | 0.33 | 0.35 |
| Price value | PV1 | 0.32 | 0.03 | 0.05 | 0.00 | -0.10 | 0.29 | 0.23 | 0.39 | 0.37 | 0.23 | **0.91** | 0.29 |
|  | PV2 | 0.34 | 0.00 | 0.05 | 0.00 | -0.06 | 0.36 | 0.28 | 0.46 | 0.44 | 0.35 | **0.96** | 0.32 |
|  | PV3 | 0.37 | -0.02 | 0.05 | -0.04 | -0.06 | 0.35 | 0.26 | 0.47 | 0.42 | 0.34 | **0.95** | 0.35 |
| Social influence | SI1 | 0.42 | 0.13 | -0.06 | -0.04 | 0.01 | 0.24 | 0.24 | 0.55 | 0.27 | 0.32 | 0.32 | **0.97** |
|  | SI2 | 0.43 | 0.15 | -0.07 | -0.06 | 0.01 | 0.24 | 0.24 | 0.55 | 0.31 | 0.31 | 0.33 | **0.98** |
|  | SI3 | 0.44 | 0.13 | -0.08 | -0.07 | 0.03 | 0.24 | 0.25 | 0.56 | 0.34 | 0.32 | 0.34 | **0.98** |
